# SLAMF6 is associated with the susceptibility and severity of rheumatoid arthritis in the Chinese population

**DOI:** 10.1186/s13018-021-02901-9

**Published:** 2022-01-11

**Authors:** Guodong Xia, Yetian Li, Wei Pan, Chengmei Qian, Lin Ma, Jingli Zhou, Henggui Xu, Chen Cheng

**Affiliations:** 1 Department of Orthopaedic Surgery, The JiangYan TCM Hospital of Taizhou City, JiangYan Road No. 699, Taizhou City, 225500 China; 2 Department of Orthopaedic Surgery, The First Affiliated Hospital of Anhui Medical University, Hefei City, China; 3 Department of Orthopaedic Surgery, The Huai’an Second People’s Hospital, Huai’an City, China

**Keywords:** Rheumatoid arthritis, Susceptible, SLAMF6, Severity

## Abstract

**Objectives:**

A recently published genome-wide association study identified six novel loci associated with rheumatoid arthritis (RA) in Korean population. We aimed to investigate whether these newly reported RA-risk loci are associated with RA in the Chinese population and to further characterize the functional role of the susceptible gene.

**Methods:**

The susceptible variants of RA were genotyped in 600 RA patients and 800 healthy controls, including rs148363003 of *SLAMF6*, rs117605225 of *CXCL13*, rs360136 of *SWAP70*, rs111597524 of *NFKBIA*, rs194757 of *ZFP36L1* and rs1547233 of *LINC00158*. Synovial tissues were collected from the knee joint of 50 RA patients and 40 controls without osteoarthritis for the gene expression analysis. Inter-group comparisons were performed with the Chi-square test for genotyping data or with Student's t-test for gene expression analysis.

**Result:**

For rs148363003 of *SLAMF6*, RA patients were observed to have a significantly lower frequency of genotype CC (4.5% vs. 0.9%, *p* = 0.004) as compared with the controls. The frequency of allele C was remarkably higher in the patients than in the controls (11.5% vs. 8.0%, *p* = 0.002), with an odds ratio of 1.49 (95% CI = 1.16–1.92). There was no significant difference between the patients and the controls regarding genotype or allele frequency of the other 5 variants. The mRNA expression of *SLAMF6* was 1.6 folds higher in the RA patients than in the controls. Moreover, *SLAMF6* expression was 1.5 folds higher in patients with genotype CC than in the patients with genotype TT.

**Conclusions:**

*SLAMF6* was associated with both the susceptibility and severity of RA in the Chinese population. Moreover, rs148363003 could be a functional variant regulating the tissue expression of *SLAMF6* in RA patients. It is advisable to conduct further functional analysis for a comprehensive knowledge on the contribution of this variant to the development of RA.

## Introduction

Rheumatoid arthritis (RA) is a complex autoimmune disorder characterized by destruction of cartilage and joint caused by inflammatory synovitis [1, 2]. RA usually develops between 40 and 50 years of age. The prevalence of the disease was estimated to be 0.5–1% among the world population and was more frequently developed in women than in men [3–5]. Although the etiology of RA is yet to be uncovered, earlier studies demonstrated that both environmental and genetic risk factors might play a major role in the onset and progression of the disease [6–8]. Previous familial studies indicated the risk of RA was remarkably higher in subjects with a familial history of RA, which was likely to be attributed to both inherited and environmental factors [9, 10]. The overall heritability of RA has been estimated to be about 50–65% by twin studies [11, 12]. Apparently, a combination between genetic background and environmental triggers confers an increased risk for RA.

To date, numerous efforts have been undertaken to identify specific genes and pathways implicated in the development of RA. Genome-wide association studies (GWAS) have discovered more than 100 RA-associated genetic variants in multiple ancestries [13–17]. Julia et al. [18] analyzed 317,503 SNPs in 400 RA patients and 400 controls and identified *KLF12* as a new susceptibility gene for RA in the Spanish population. Saxena et al. [19] tested > 7 million SNPs in a total of 511 RA cases and 352 healthy controls and reported 2 novel associations specific to Arab populations at the 5q13 and 17p13 loci. According to the most recent studies, previously reported loci were able to explain only about 15% of the total risk in susceptibility to RA [15]. Considering substantial missing fraction of heritability, continuous effort in identifying additional RA variants is thus necessary to better understand the disease etiology.

In a recently published meta-analysis of GWAS for 4068 RA cases and 36,487 healthy controls, Kwon et al. [15] identified six novel RA-risk loci that reached the genome-wide significance in Korean population. It is now well established that for GWAS findings in complex disease genetics, one of the pivotal tasks is to further replicate genetic changes in different populations and link these susceptible variants to potential gene functions. In this study, we aimed to investigate whether the newly reported RA-risk loci were associated with RA in the Chinese population and to further characterize the functional role of the susceptible gene.

## Methods

### Subjects

Under the approval of the Ethics Committee of our institutions, a cohort of 600 RA patients who visited our center for treatment between June 2014 and October 2020 were enrolled in this study. All the patients were from Chinese Han population, who were diagnosed as RA according to the 2010 American College of Rheumatology criteria for RA classification [20]. The 28-Joint Count Disease Activity Score (DAS28) was used to assess the disease activity for each patient [21, 22]. Patients with DAS28 > 3.2 were classified as active RA, and patients with DAS28 ≤ 3.2 were classified as inactive RA. Eight hundred age-matched healthy subjects were recruited as normal controls. The baseline characteristics of RA patients were collected from the medical records, including age, gender, body mass index (BMI), the serum level of rheumatoid factor (RF), erythrocyte sedimentation rate (ESR) and C-reactive protein (CRP).

### Genotyping of target SNPs

With the informed consent obtained from the subjects, blood samples were then collected for genomic DNA extraction using the commercial kit (QIAGEN, Tokyo, Japan), which were then stored at − 20 °C. Approximately 10 ng of the DNA sample was used for genotyping of the following 6 SNPs with TaqMan SNP Genotyping Assay, including rs148363003 of *SLAMF6*, rs117605225 of *CXCL13*, rs360136 of *SWAP70*, rs111597524 of *NFKBIA*, rs194757 of *ZFP36L1* and rs1547233 of *LINC00158*. Amplification was performed in 20 μl reaction volumes, containing 2 μl of TaqMan genotyping assay mix, 6 μl of genomic DNA, 2 μl of distilled deionized water and 10 μl of the TaqMan genotyping master mix. The interpretation of genotyping assay results was read on ABI 7900HT sequence detection system (Applied Biosystem, Foster City, CA). Twenty percent of the tests were randomly repeated to validate the reproducibility of the genotyping results.

### Gene expression analysis of SLAMF6

During total knee replacement surgery, samples of synovial tissues were collected from the knee joint of 50 RA patients. Forty patients undergoing surgery for cruciate ligament rupture or meniscus tear were recruited as the control group. The mean age of the RA patients was 44.6 ± 11.9 years and their mean disease duration was 52.1 months (range 19–129 months). The mean ages of the control group were 42.2 ± 9.5 years. The total RNA was extracted from synovial tissues using Trizol reagent (QIAGEN, Tokyo, Japan) according to the standard protocol, which was then reversely transcribed by the PrimeScriptRT Master Mix kit (TaKaRa, Tokyo, Japan). The mRNA expression of *SLAMF6* was quantified by quantitative real-time PCR (qPCR) with glyceraldehyde-3-phosphate dehydrogenase (GAPDH) used as internal controls. The gene-specific primers were as follows, forward 5′- GAGTCCGCAAGGAACCTAGAG-3′, reverse 5′-TCCCTGTTTGAATGAGTGACTGA-3′ for POC5, and forward 5′-GAGTCAACGGATTTGGTCGT-3′, reverse 5′-TTGATTTTGGAGGGATCTCG-3′ for GAPDH that functions as endogenous control. All amplification reactions were carried out in triplicate.

### Statistical analysis

For continuous descriptive data, the results were displayed as the mean ± standard deviation (SD). The Hardy–Weinberg equilibrium (HWE) method was used to examine the genotype frequency of the normal controls. The Chi-square analysis was used to compare the frequency of genotype and risk allele between the RA cases and controls, with the odds ratio (OR) and 95% confidential intervals (CIs) calculated for each SNP. Inter-group comparison of the expression of *SLAMF6* was assessed by the Student’s t-test. Correlations between *SLAMF6* expression and serum indexes were analyzed by Spearman’s correlation analysis. The SPSS software (version 23.0, Chicago, USA) was used for statistical analysis. A *P* value of less than 0.05 was considered statistically significant.

## Results

### Demographic data of the subjects

The baseline clinical characteristics of the subjects are summarized in Table 1. For association analysis, there was no significant difference between the patients and the controls regarding the mean age (43.4 ± 12.9 vs. 42.6 ± 13.4, *p* = 0.26), BMI (24.1 ± 5.6 vs. 24.4 ± 4.9, *p* = 0.29) or gender (*p* = 0.22). The mean ESR level was 21.1 ± 11.9 mm/h in RA patients. The mean value of RF and CRP was 84.8 ± 7.8 (IU)/ml and 44.3 ± 19.7 mg/L, respectively.

**Table 1:** Baseline characteristics of the subjects

	Patients (*n* = 600)	Controls (*n* = 800)	*P*
Age (years)	43.4 ± 12.9	42.6 ± 13.4	0.26
Gender (male/female)	192:408	281: 519	0.22
BMI (kg/m^2^)	24.1 ± 5.6	24.4 ± 4.9	0.29
DAS28	3.5 ± 1.6	N/A	N/A
ESR (mm/h)	21.1 ± 11.9	N/A	N/A
RF (IU/ml)	84.8 ± 7.8	N/A	N/A
CRP (mg/L)	44.3 ± 19.7	N/A	N/A

### Replication of RA-associated variants

HWE test revealed no remarkable difference regarding the genotype frequency among the controls. As shown in Table 2, for rs148363003 of *SLAMF6*, RA patients were observed to have a significantly higher frequency of genotype CC (4.5% vs. 0.9%, *p* = 0.004) as compared with the controls. The frequency of allele C was remarkably higher in the patients than in the controls (11.5% vs. 8.0%, *p* = 0.002), with an odds ratio of 1.49 (95% CI = 1.16–1.92). There was no significant difference between the patients the controls regarding genotype or allele frequency of the other 5 variants.

**Table 2:** Comparison of the genotype and allele frequency between the patients and controls

		Genotype			*P*	Allele		*P*	Odds ratio (95% CI^a^)
		XX	Xx	xx		X	x		
rs148363003	Patients (*n* = 600)	489 (81.5%)	84 (14.0%)	27 (4.5%)	0.004	1062 (88.5%)	138 (11.5%)	0.002	1.49 (1.16–1.92)
	Controls (*n* = 800)	679 (84.9%)	114 (14.2%)	7 (0.9%)		1472 (92.0%)	128 (8.0%)		
rs117605225	Patients (*n* = 600)	570 (95.0%)	26 (4.3%)	4 (0.7%)	0.54	1166 (97.2%)	34 (2.8%)	0.52	0.87 (0.56–1.34)
	Controls (*n* = 800)	750 (93.8%)	48 (6.0%)	2 (0.2%)		1548 (96.7%)	52 (3.3%)		
rs360136	Patients (*n* = 600)	180 (30.0%)	291 (48.5%)	129 (21.5%)	0.92	652 (54.3%)	548 (46.7%)	0.95	1.01 (0.86–1.16)
	Controls (*n* = 800)	234 (29.2%)	403 (50.4%)	163 (20.4%)		871 (54.4%)	729 (46.6%)		
rs111597524	Patients (*n* = 600)	301 (50.2%)	280 (46.6%)	19 (3.2%)	0.71	882 (73.4%)	318 (26.6%)	0.74	0.97 (0.82–1.15)
	Controls (*n* = 800)	388 (48.5%)	391 (48.9%)	21 (2.6%)		1167 (72.9%)	433 (27.1%)		
rs194757	Patients (*n* = 600)	284 (47.4%)	256 (42.6%)	60 (10.0%)	0.18	824 (68.7%)	376 (31.3%)	0.22	0.91 (0.77–1.06)
	Controls (*n* = 800)	351 (43.9%)	362 (45.3%)	87 (10.9%)		1064 (66.5%)	536 (33.5%)		
rs1547233	Patients (*n* = 600)	332 (55.3%)	244 (40.8%)	24 (4.0%)	0.20	908 (75.6%)	292 (24.4%)	0.37	0.92 (0.78–1.09)
	Controls (*n* = 800)	423 (52.9%)	332 (41.5%)	45 (5.6%)		1187 (73.7%)	413 (26.4%)		

XX, Xx and xx indicated the homogeneous of the major allele, heterogeneous of the major and the minor allele and homogeneous of the minor allele, respectively. X and x indicated wild-type allele and mutant allele, respectively

### Tissue expression of the SLAMF6

**Fig. 1:**
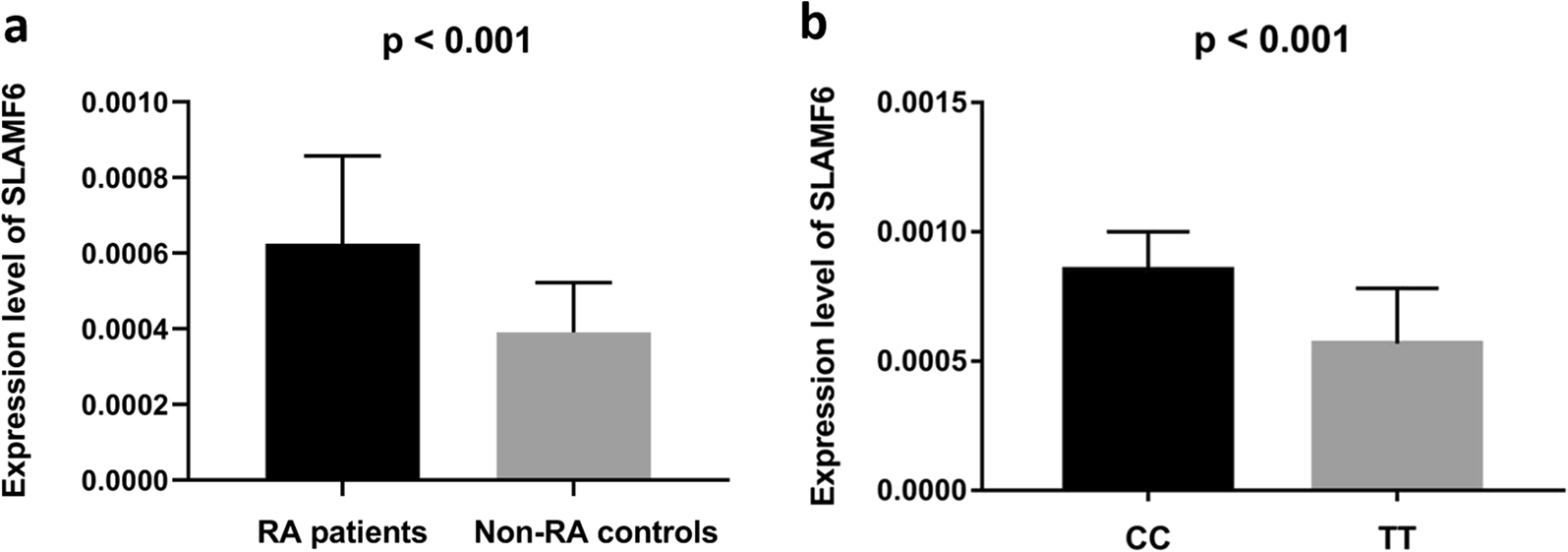
Tissue expression of *SLAMF6* in RA patients. **a** RA patients were found to have remarkably increased expression of *SLAMF6* gene as compared with the controls. **b** The mRNA expression of *SLAMF6* was 1.5 folds higher in patients with genotype CC than in the patients with genotype TT

A total of 50 RA patients and 40 non-RA controls were included in expression analysis. The two groups were matched in terms of age (44.6 ± 11.9 years vs. 42.2 ± 9.5 years, *p* = 0.29) and BMI (24.8 ± 3.6 vs. 24.1 ± 3.9, *p* = 0.37). As illustrated in Fig. 1A, the mRNA expression of *SLAMF6* was 1.6 folds higher in the RA patients than in the controls (0.000625 ± 0.0000232 vs. 0.000390 ± 0.000132, *p* < 0.001). For RA patients, there were 40 cases with genotype TT of rs148363003 and 10 cases with genotype CC. As shown in Fig. 1B, the mRNA expression of *SLAMF6* was 1.5 folds higher in patients with genotype CC than in the patients with genotype TT (0.000853 ± 0.000148 vs. 0.000568 ± 0.000214, *p* < 0.001).

### Relationship between SLAMF6 and clinical phenotypes of RA

**Fig. 2:**
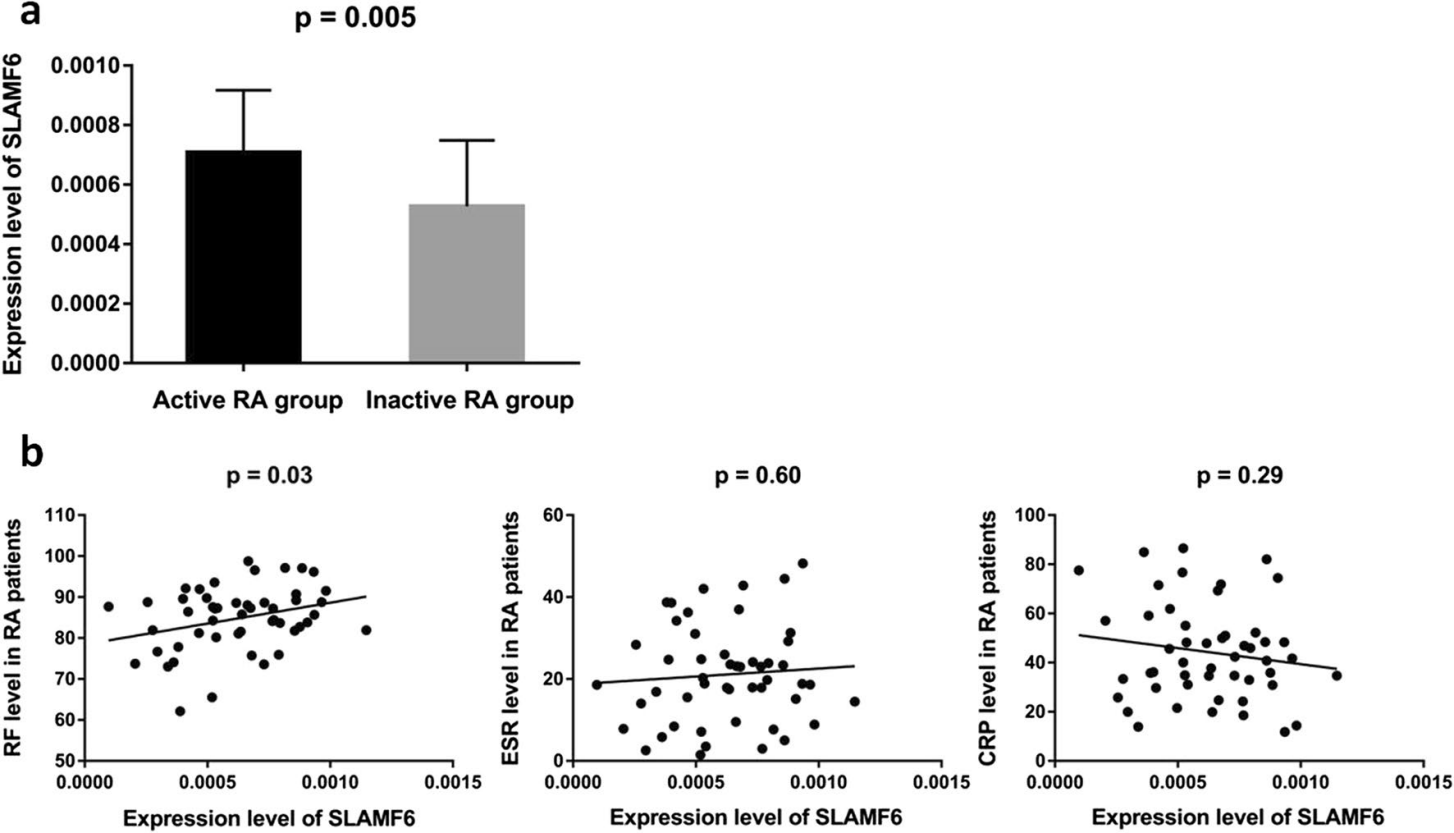
The relationship between *SLAMF6* expression and severity of RA. **a** Patients in active RA group (*n* = 27) had significantly higher *SLAMF6* expression than patients in inactive RA group (*n* = 23). **b** The mRNA expression level of *SLAMF6* was remarkably correlated with serum RF (*r* = 0.30, *p* = 0.03). No significant correlation with ESR (*r* = 0.08, *p* = 0.60) or CRP (*r* = − 0.15, *p* = 0.29) was found

Of the 50 RA patients included in tissues analysis, 27 patients had DSA28 of more than 3.2, who were thus assigned to active RA group. Inter-group comparison showed that patients in active RA group (*n* = 27) had significantly higher *SLAMF6* expression than those in inactive RA group (*n* = 23) (0.000708 ± 0.000210 vs. 0.000527 ± 0.000222, *p* = 0.005) (Fig. 2a). As shown in Fig. 2b, the mRNA expression level of *SLAMF6* was remarkably correlated with serum RF (*r* = 0.30, *p* = 0.03). As for ESR (*r* = 0.08, *p* = 0.60) and CRP (*r* = − 0.15, *p* = 0.29), no significant correlation with *SLAMF6* expression was found.

## Discussion

As recent meta-analysis of GWAS data in Korean cohorts identified 6 novel loci of RA [15], we replicated these variants in the Chinese RA patients and confirmed that rs148363003 of *SLAMF6* was associated with RA in the Chinese population. For rs148363003, allele T was a wild-type allele, and allele C was a mutant allele. Genotype CC was indicative of remarkably higher risk of RA. We observed that allele C of rs148363003 can remarkably add to the risk of RA by 1.49 folds, which was consistent with an increased RA risk of 1.62 folds in the Korea population as reported by Kwon et al. [15]. The rs148363003 is located at 11 kb downstream of *SLAMF6*. Through expression quantitative trait locus mapping based on public database, Kwon et al. [15] reported that rs148363003 had chromatin interactions with 13 coding genes on chromosome 1. To further clarify the biological basis underlying the association between rs148363003 and the risk of RA, we preliminarily investigated the relationship between rs148363003 and tissue expression of *SLAMF6*. For the first time, we analyzed the expression of *SLAMF6* gene in the synovial tissues of RA patients as well as in the controls. Remarkably higher tissue expression of *SLAMF6* was observed in RA patients than in the controls. Moreover, patients with genotype CC of rs148363003 were found to have remarkably increased expression of *SLAMF6* than those with genotype TT. Since the frequency of genotype CC was remarkably higher in patients than in normal controls, it was plausible that the mutant allele C added to the risk of RA via regulation of *SLAMF6*. Interestingly, as shown in HaploReg database [23], rs148363003 is predicted to alter transcription factor-binding sites of *SLAMF6*. A fine-mapping of the genomic region around rs148363003 is warranted to further elucidate its regulatory role in the tissue expression of *SLAMF6*.

As reported in previous studies, the susceptible genes of RA could be associated with the severity of the disease [24–26]. Elkhawaga et al. [24] reported that rs28362491 of NFKB1 contributed to both susceptibility and severity of RA. Zhao et al. [25] reported that rs1801278 of IRS-1 was associated with increased risk and disease activity of RA. In this study, the relationship between *SLAMF6* and RA activity was also analyzed. Patients with high RA activity were found to have significantly higher *SLAMF6* expression than patients with low RA activity. Moreover, there was significant correlation between *SLAMF6* expression and serum RF. Collectively, *SLAMF6* may act as a modifier gene which is prognostic of the progression of RA.

To date, the exact function of *SLAMF6* in RA remains obscure. The product of *SLAMF6* is a member of superfamily immunoglobulin (Ig) domain-containing molecules that play a role in interactions between naive lymphocytes. Previous functional studies indicated that the *SLAMF6* could be involved in a series of inflammatory diseases [27–29]. Wang et al. [27] reported that *SLAMF6* might function as an inhibitory receptor that regulates autoimmune responses. Savola et al. [29] reported accumulation of mutations in *SLAMF6* in expanded T cells may have pathogenic significance for RA. Other members of SLAMF have been reported to contribute to the accumulation of antibody producing cells in patients with systemic lupus erythematosus, which therefore were considered as potential therapeutic targets for autoimmune diseases [30]. In the current study, we preliminarily observed that the tissues expression of *SLAMF6* was associated with RA activity. Further investigation into the relationship between serum soluble *SLAMF6* concentration and clinical characteristics of RA patients is warranted to better decipher the potential application of SLAMF6 as a clinical biomarker for RA.

In the current study, no association between the 5 SNPs and RA in the Chinese population was found in the current study. However, we still cannot entirely exclude the association of these 5 genes with RA. Several limitations of this study should be addressed here. First, due to the inherent defect of retrospective research, the lack of replication was probably attributed to the relatively small sample size of current study. Larger sample size may help yield more convincing outcome. Second, we used patients with cruciate ligament rupture or meniscus tear as control group for gene expression of *SLAMF6* in synovial tissue. It was difficult to collect synovial tissue from healthy population. The third limitation of the present study lies in that no in vivo experiment was performed to clarify the regulatory role of rs148363003 in *SLAMF6* expression. Further functional experiments are therefore necessary to clarify the underlying regulatory mechanism.

## Conclusions

*SLAMF6* was associated with both the susceptibility and severity of RA in the Chinese population. Moreover, rs148363003 could be a functional variant regulating the tissue expression of *SLAMF6* in RA patients. It is advisable to conduct further functional analysis for a comprehensive knowledge on the contribution of this variant to the development of RA.

## Data Availability

All data used in this study are available at the request of editors, reviewers and the research community.

## Abbreviations

RA: Rheumatoid arthritis
GWAS: Genome-wide association studies
DAS28: 28-Joint Count Disease Activity Score
BMI: Body Mass Index
RF: Rheumatoid factor
ESR: Erythrocyte sedimentation rate
CRP: C-reactive protein
HWE: Hardy–Weinberg equilibrium
OR: Odds ratio
CIs: Confidential intervals
